# The Journal—Vol. XXVII.

**Published:** 1886-08

**Authors:** 


### Editorial.

### *THE JOURNAL—VOL. XXVII.*

With this number, the JOURNAL enters on the twenty-seventh year of its existence, and the eighth under the control of its present editors. As we look back upon these last seven years, we realize that though our labor has been arduous, there has been much to make that labor a pleasure, and that our efforts to do our duty as medical journalists, have met with a most gratifying recognition from our readers, and, consequently, have not been barren of results. The standard of medical education has, in Western New York, been advanced to a degree which, without legislation, few supposed possible. The attendance upon the meetings of the medical societies, and the quality as well as the number of papers presented, have shown a steady growth in medical culture.

The quackish custom, too common in some other cities, of physicians seeking notoriety by allowing highly colored reports of cases to appear in the daily papers, has not taken root here.

There has, alas, been too much of strife and discord in the profession, but for that we are not responsible, except in so far as the upholding of principle against strong opposition may have led to it; but the profession will, in the end, be the purer and stronger for such conflicts as these!

We would be ungrateful to our kind friends, did we not express our sincere appreciation of their cordial support, especially during the year past. The result has been a year of unparalleled prosperity in the history of the JOURNAL. The number of our subscribers, both in the city and country, has materially increased. Subscriptions have been more promptly paid, and we have thus been enabled to increase the number of our pages, to illustrate freely, and to give our readers, as many of them have been kind enough to say, a better journal than ever before.

For the ensuing year, we have no promises to make; indeed, prospective pledges have of late been monopolized by the new journals which are launched with mighty promises. The ridicule which commonly follows upon the performance, tends always to the better appreciation of old and tried journals. Our readers—judging our future by our past—know that we will not go backwards.

As an indication of this, we call attention to this first number of the volume. It will be noticed that selections, society proceedings, etc., are printed in smaller type, thus, practically, adding about six pages to the JOURNAL. Several other minor changes have been made, which, we hope, will be acceptable to our readers.

The editors take this opportunity to give a due meed of praise to the energetic and accomplished physicians whose labors, as associate editors, during the year past, has added so much of interest to our pages, and it is but just, too, to give our especial thanks to Dr. Crego, who has contributed largely to the editoral work, and to Dr. Campbell, whose admirable translations from foreign journals have proved both valuable and interesting.

We are glad to announce that the editorial staff will remain the same during the ensuing year.

